# Directional coupling of surface plasmon polaritons at complementary split-ring resonators

**DOI:** 10.1038/s41598-019-43914-z

**Published:** 2019-05-14

**Authors:** Yongsop Hwang, Jin-Kyu Yang

**Affiliations:** 1 Institute of Application and Fusion for Light, Kongju National University, Cheonan, 31080 South Korea; 2 Department of Optical Engineering, Kongju National University, Cheonan, 31080 South Korea

**Keywords:** Nanophotonics and plasmonics, Sub-wavelength optics

## Abstract

We propose a complementary split-ring resonator (CSRR) for a directional coupling of surface plasmon polaritons. An air-slot split-ring in a gold film is investigated using the finite-difference time-domain method. The normally incident light couples to either a monopole or a dipole SPP depending on the polarization of light. Adjusting the angle of the linear polarization of the incident light enables a one-way propagation of SPPs on the gold film. Theoretical analysis based on the propagation of cylindrical waves from the SPP point source is provided with Hankel function. The propagated power in one direction is obtained to be 30 times higher than the opposite direction with a coupling efficiency of 18.2% from the simulation for an array of the CSRRs. This approach to the directional coupling of SPPs will be advantageous for miniaturizing photonic and plasmonic circuits and devices.

## Introduction

Surface plasmon polaritons (SPPs) are the collective oscillations of electrons which propagate along the interface between a metal and a dielectric^1^. SPPs contain some unique properties such as strong light confinement beyond diffraction limit^2^ and high sensitivity to the surrounding materials^3^, which have led advances in subwavelength optics^4–6^, photonic information^7,8^, optoelectronics^9,10^ and quantum information^11,12^. Their linear and planar propagation characteristics in one- and two-dimensional metallic structures, respectively, have been investigated to reduce the size of photonic devices and improve the propagation efficiency^4,6,13,14^. Coupling structures are often implemented primarily to overcome the momentum mismatch between the free space incident light and the guided modes of SPPs^15^. The coupling structures can also perform an additional function such as polarization selective directional coupling.

Exciting SPPs to propagate in the desired direction is essential for diverse practical applications using ultracompact optical components^16^. One of the common schemes for SPP excitation on a metallic plane is the normal incidence of a polarized beam. Controlling the excited SPPs in a nanophotonic device based on the polarization state of the normally incident beam is beneficial for photonic data processing and computation. Therefore, polarization selective directional couplers have been suggested and demonstrated for circular^17–19^ and linear polarizations^20,21^ by exciting dipole SPPs at nanoslots which are periodically arranged to make constructive interference for propagation in the desired direction. By placing two slots with the adjusted angle and separation, a directional coupling for the circular polarization was demonstrated^17^. On the other hand, an array of nanorods which support localized surface plasmons can be arranged as an antenna array to realize unidirectional coupling of the desired linear polarization^20–23^. This approach using the interference of dipole SPPs from two separate sources requires two nanoslots.

Here, we propose a different approach of using the interference of a *monopole* and a *dipole* SPPs excited at a complementary split-ring resonator (CSRR). A split-ring resonator (SRR) which has a short disconnection at one position of the ring is particularly known for the negative refractive index generation at microwave regime^24,25^. A CSRR is an inverted counterpart of an SRR which is a split-ring of an air slot perforated on a metallic film. The CSRRs have been investigated for guided modes in left-handed waveguides^26^, realizing zero refractive index^27^, filtering a frequency band coupled to a waveguide^28^, beaming microwave SPPs^29^, interacting with spoof SPPs^30^ and tuning the filtering wavelength^31^. We employed a CSRR to support SPPs of both monopole and dipole radiation characteristics, whose interference results in a one-way propagation. Differently from most previous directional couplers employing dipole SPPs^17–23^, the hybrid state of a monopole and a dipole SPPs inherently holds the directionality. We theoretically analyzed the one-way propagation based on the travelling cylindrical waves which are represented by Hankel function. The proposed CSRR is advantageous to miniaturize a directional coupler in SPP based photonic integrated circuits since it is a single subwavelength structure which supports both the monopole and the dipole SPP radiations.

## SPP Modes in the Single CSRR

**Figure 1:**
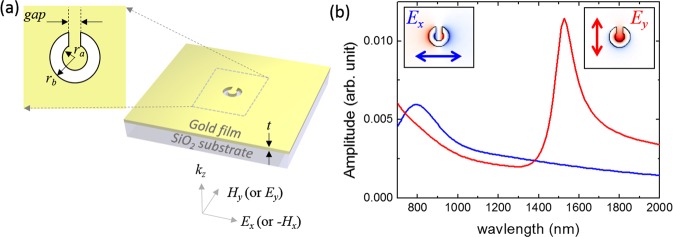
(**a**) Schematic diagram of SPP excitation at the CSRR structure and (**b**) spectral response according to the input polarization direction. The blue and the red lines indicate the *x*- and *y*-polarized incidences, respectively. The inset in (**a**) shows the structural parameters and the insets in (**b**) show the *z*-component of the electric-field (*E*_*z*_) distributions of the excited SPP modes.

The resonant SPP modes were numerically investigated by the finite-difference time-domain (FDTD) method^32^ using a homemade code. Figure 1(a) shows the schematic diagram of the SPP excitation at the CSRR air slot structure with 50-nm-thick Au film^33^ on the SiO_2_ substrate (*n* = 1.55). The absorption boundary was applied for all three directions. The inner (*r*_*a*_) and the outer (*r*_*b*_) radii of the CSRR are 40 nm and 90 nm, respectively. The gap size is 50 nm. A plane wave polarized in either *x*- or *y*-direction is normally incident to the substrate. Figure 1(b) shows the resonant spectra according to the input polarization directions. A strong resonance was found at the wavelength of 1525 nm for the incident light polarized in *y*-direction which is the symmetric axis of the CSRR, while the *x*-polarized source induces a resonance at 795 nm. The plasmonic modes excited by *x*- and *y*-polarized sources have *E*_*z*_-field profiles which are anti-symmetric and symmetric about the symmetric axis of the CSRR, respectively, as shown in the insets of Fig. 1(b). The *E*_*z*_-field is depicted since this field component is dominantly related to the in-plane propagation of the SPPs. The anti-symmetric and symmetric characteristics of the excited SPP modes are essential for the directional coupling.

**Figure 2:**
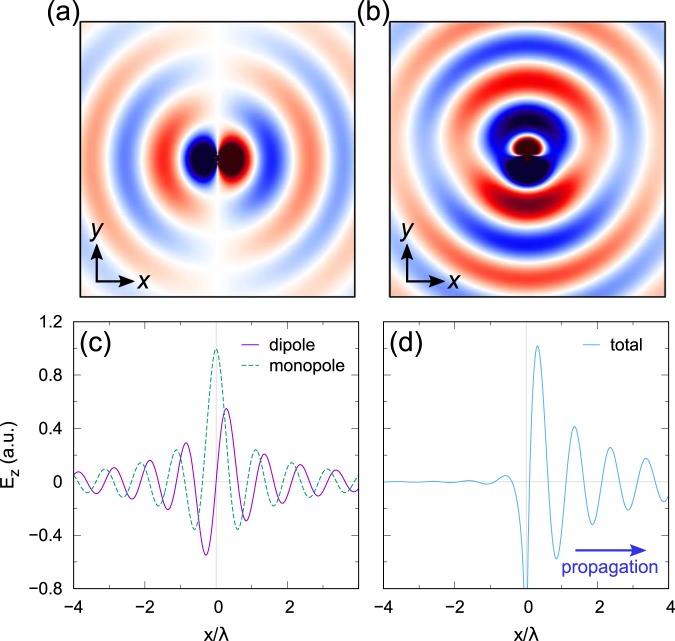
The numerically simulated *E*_*z*_-field distributions of (**a**) a dipole and (**b**) a monopole SPP sources. The *E*_*z*_-field amplitudes of the cylindrical waves from (**c**) a dipole and a monopole sources and (**d**) the total wave which shows a one-way propagation as a result of their interference.

Figure 2(a,b) show the numerically simulated *E*_*z*_-field distributions of the SPPs along the interface between Au and air excited by *x*- and *y*-polarized incident waves, respectively, at λ = 1475 nm which is the peak wavelength of the scattering cross section at the *y*-polarized incidence. At the *x*-polarized input light, the coupled SPP mode has antisymmetric *E*_*z*_-field distribution radiated from a point-like dipole source as shown in Fig. 2(a). However, at the *y*-polarized input light, the coupled SPP has a symmetric *E*_*z*_-field distribution from a point-like monopole source (Fig. 2(b)). The radiated electric field amplitude from a monopole point source is mathematically represented as

1 $${E}_{z}^{m}(\rho ,t)={E}_{0}^{m}{H}_{0}^{(1)}(k\rho ){e}^{-i{\rm{\omega }}t-{\rm{\gamma }}\rho },$$

where ${E}_{0}^{m}$ is the initial *E*_*z*_-field amplitude at the source point, and *k* and *ω* are the wavevector and the frequency of the wave, respectively. It is a radially travelling cylindrical wave described by the zeroth order Hankel function of the first kind ${H}_{0}^{(1)}$ whose amplitude decreases by $1/\sqrt{\rho }$ when $\rho \gg 1$ which obeys the law of conservation of energy^34^. The propagation loss due to the energy dissipation of SPPs is represented by the attenuation coefficient γ. The dipole counterpart is represented as

2 $${E}_{z}^{d}(\rho ,\varphi ,t)={E}_{0}^{d}{H}_{1}^{(1)}(k\rho ){e}^{i\varphi }{e}^{-i\omega t-\gamma \rho },$$

where ${H}_{1}^{(1)}$ is the first order Hankel function of the first kind and *ϕ* is the azimuthal angle with which the anti-symmetric characteristic of the dipole radiation is expressed by *e*^*iϕ*^. The propagation patterns of the SPPs from the monopole and the dipole sources are shown in Fig. 2(c). The attenuation coefficient γ is set to be 1/(5λ_SPP_) based on the reported propagation lengths of guided plasmonic modes^13,35,36^. Subsequently, the interference of the monopole and the dipole radiations can be obtained by the linear superposition of the Eqs. (1) and (2) as ${E}_{z}(\rho ,\varphi ,t)={E}_{z}^{m}(\rho ,t){e}^{-i\delta }+{E}_{z}^{d}(\rho ,\varphi ,t)$ where *δ* is the temporal phase difference between the monopole and the dipole modes due to the off-resonance excitation. The total *E*_*z*_-field at *t* = 0 is depicted assuming ${E}_{0}^{m}={E}_{0}^{d}=1$ with the phase difference *δ* of *π*/2 in Fig. 2(d). It is obvious that the interference of the two radiations results in a one-way propagation (for example, in positive *x*-direction) when the initial amplitudes of the monopole and the dipole are the same and a proper temporal phase difference is given. In order to obtain the one-way propagation of SPPs, the ratio of the two initial amplitudes can be adjusted simply by controlling the polarization angle of the incident wave. Travelling patterns of the monopole and the dipole waves as well as the total field at different time are presented in Fig. S3 in Supplementary Information.

### Unidirectional Propagation of SPPs in Array of CSRRs

**Figure 3:**
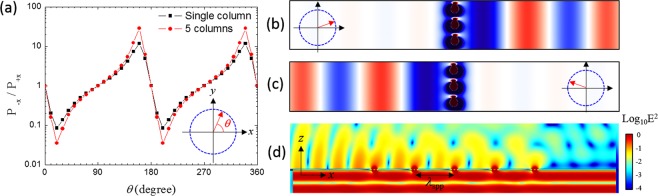
(**a**) Power ratio of the coupled SPPs propagating in the negative to the positive *x-*direction, *P*_−*x*_/*P*_+*x*_, as a function of the polarization angle *θ* of the incidence light in arrays of the CSRRs. The single-column and 5-column arrays are shown in the left inset. (**b**) The electric field distributions of the coupled SPPs from the single-column array at *θ* = 20° and (**c**) at *θ* = 160°. (**d**) The cross-sectional profile of the electric field intensity distribution from the 5-column array at *θ* = 20° on a logarithmic scale.

Based on the understanding of the SPP propagation from a single CSRR, we numerically investigated the unidirectional SPP coupling using arrays of the CSRRs by utilizing the different symmetry properties of the SPP modes depending on the polarization directions of the incident light. A single-column array of the CSRRs is composed by placing the CSRR along its symmetric axis with the lattice period of 300 nm. Similarly, a five-column array consists of 5 single-column arrays with the period of 1465 nm in the *x*-axis. Figure 3(a) shows the FDTD results of the power ratio of the coupled SPPs propagating in the negative to the positive *x*-direction, *P*_−*x*_/*P*_+*x*_, as a function of the linearly polarized angle *θ* with respect to the +*x*-direction as shown in its inset. Here, the vacuum wavelength of the input plane wave is set to be 1475 nm, the peak wavelength of the power flux at the *y*-polarized light (See Fig. S1(d) in Supplementary Information). At *θ* = 20°, the power flux of the SPP propagating along the +*x*-direction is ~10 times higher than that along the −*x*-direction. At *θ* = 160°, on the other hand, the power flux of the SPP propagating along the −*x*-direction is ~10 times higher than that along the +*x*-direction. The *E*-field profiles at *θ* = 20° and 160° depicted in Fig. 3(b,c), respectively, clearly demonstrate the characteristics of the directional coupling. As the number of columns of the CSRRs with the spacing of λ_spp_ (1465 nm) increases, the unidirectional SPP coupling is more pronounced due to the constructive interference. Figure 3(a) shows that the power ratio of the 5-column array is obtained to be ~30 at *θ* = 160° which is 3 times higher than the single-column array. The cross-sectional profile of the electric field intensity from the 5-column array at *θ* = 20° is shown in Fig. 3(d). The one-way propagation of the coupled SPP in positive *x*-direction is clearly observed. At the single-column array, the Poynting flux of the monopole is obtained approximately three times greater than the dipole hence the polarization angle of 20° excites the two modes with similar amplitudes. Therefore, the maximum power ratio is obtained by the interference of the two radiations at the given angle (See Supplementary Information for details). The dependence of the wavelength of the incident light and the power ratio modulation by the phase difference between the two SPPs are also discussed in Supplementary Information.

**Figure 4:**
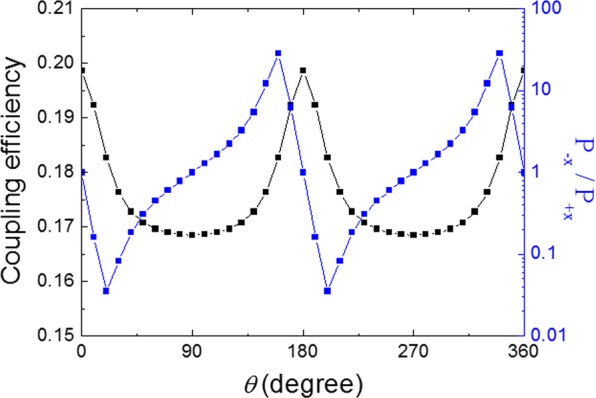
Coupling efficiency (black) and power ratio (blue) of the 5-column array of the CSRRs as functions of the polarization angle.

High coupling efficiency is required for a directional coupler to be practically applied. The coupling efficiency of the five-column array is obtained as a function of the polarization angle of the incident light as shown in Fig. 4. The coupling efficiency is defined as the ratio of the transmitted power at the top surface of the gold film to the propagated power in the +*x*- and −*x*-directions on the surface. The coupling efficiency of 18.2% is obtained for *θ* = 20° and 160° which are the polarization angles for the maximum power ratio in the positive and the negative *x*-directions, respectively. Additionally, the wavelength dependence of the coupling efficiency for the single-column array is provided with a further discussion in Supplementary Information.

## Discussion

Our CSRR generates both a monopole and a dipole of which the physical meaning is worth to be discussed. Combining two or more resonance modes is a scientific method to realize desired properties at a photonic device. For example, Fano resonances have commonly been demonstrated by combining a dipole mode and a quadrupole mode at plasmonic nanostructures^37,38^. Another example is the ideal magnetic dipole scattering by spectrally overlapping a magnetic dipole resonance with an anapole mode^39^. Therefore, it is expected that the provided understanding of the hybridization of a monopole resonance and a dipole resonance occurring in our plasmonic nanostructures will not only be applied for the unidirectional coupling but also potentially enable to realize other required functions. Additionally, due to the point source nature of our structure, shaping an arbitrary wavefront of SPP which propagates in a targeted direction can be realized based on Huygens’ principle.

The efficiency of the directional coupler can be improved by further optimizing the structures to bring two resonances induced by the *x*- and *y*-polarized incidences. Numerical simulations varying the radius and the gap of the CSRR show possibility of the improvement (See Section 4 in Supplementary Information). With respect to the experimental demonstration, fabrication of the proposed structures can be executed using the conventional focused ion beam milling and the optical properties can be measured using a near-field scanning microscope or a free space optics with grating couplers introduced on the sample plane.

In conclusion, we proposed and numerically investigated a subwavelength CSRR to directionally couple the normally incident light to propagate in one direction depending on the polarization angle. The FDTD simulation shows that the CSRR supports both the monopole and the dipole radiations whose interference results in the one-way propagation. An analytic explanation of the directionality based on the travelling cylindrical waves from the monopole and the dipole which are represented by Hankel functions is provided. The power ratio of the propagations in the negative to the positive *x*-direction of the single- and the five-column arrays are obtained to be 10 and 30 times, respectively. Furthermore, the coupling efficiency of 18.2% at the polarization angles for the maximum directionality is achieved in the five-column array of CSRRs. This approach to the directional coupling by simultaneously exciting a monopole and a dipole SPPs from a single nanostructure will be beneficial for miniaturizing photonic and plasmonic circuits and devices. Compact polarization-sensitive optical systems such as ellipsometry and polarimetry can be realized based on this work.

## Supplementary information

Supplementary Information
