# Supplementary material for: Directional coupling of surface plasmon polaritons at complementary split-ring resonators
Source: Sci Rep. 2019 May 14;9:7348. doi: 10.1038/s41598-019-43914-z (PMC6517390; doi:10.1038/s41598-019-43914-z)
Supplement: Supplementary file 1 — Supplementary Information [file 41598_2019_43914_MOESM1_ESM.pdf]

Supplementary Information:

Directional coupling of surface plasmon polaritons at complementary split-ring resonators

Yongsop Hwang[1] and Jin-Kyu Yang[1,2,*]

[1]*Institute of Application and Fusion for Light, Kongju National University, Cheonan 31080, South Korea*
[2]*Department of Optical Engineering, Kongju National University, Cheonan 31080, South Korea*
Author e-mail address: jinkyuyang@kongju.ac.kr

1. *Numerical analysis of the single column CSRR array*

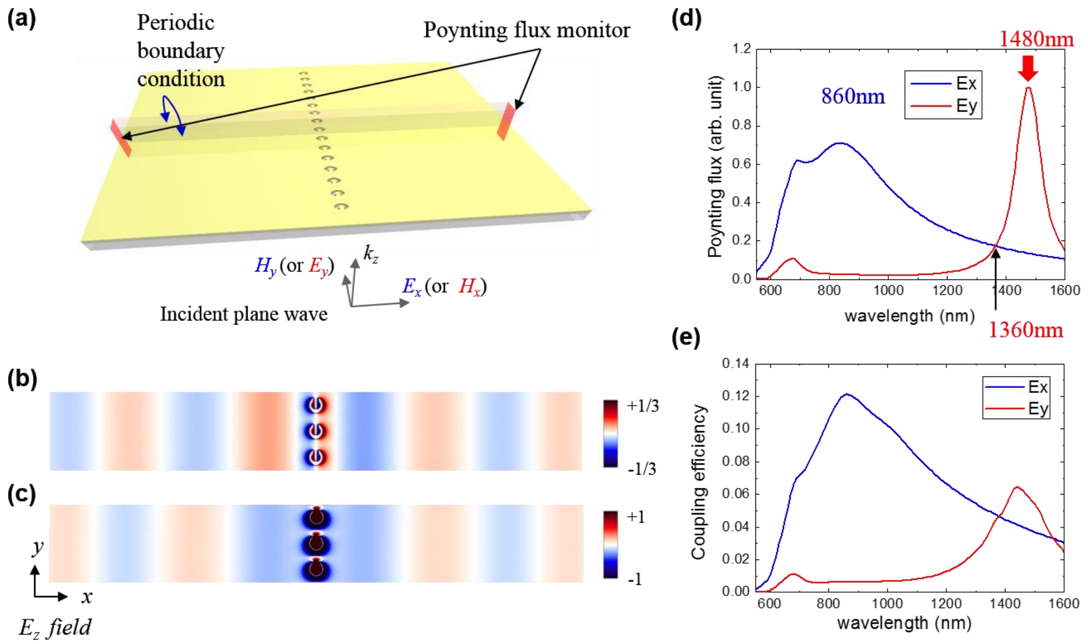

Fig. S1 (a) Schematic of the FDTD simulations of the single column CSRR array, E_z-field profiles for (b) E_x and (c) E_y incident waves at the wavelength of 1475 nm, (d) Poynting flux spectra, and (e) coupling efficiency for *x*- and *y*-polarized incident light.

A single column array of the CSRR is structured in a gold layer for the FDTD simulations as shown in Fig. S1(a). A pair of Poynting flux monitors is placed to obtain the propagated power of the SPPs. The obtained Poynting flux spectra for *x*- and *y*-polarized incident waves are shown in Fig. S1(c) where the flux ratio depends on the wavelength. The *x*- and *y*-polarizations excite SPP waves with the equal flux at the wavelength of 1360 nm while the SPP by E_y-field excitation is about 3 times greater than E_x-field at 1475 nm. The field profiles in Figs. S1(b) and (c) also show that the amplitude of the monopole radiation is approximately 3 times greater than the dipole at the given wavelength. Therefore, the power ratio for the one-way propagation can be maximized by adjusting the polarization angle θ to be 20° where the amplitudes of the monopole and the dipole propagations become equal by illuminating E_x-field which is 3 times stronger than E_y-field. Consequently, the optimum angle of polarization of the incident plane wave

for the directional coupling can be found by comparing the initial amplitudes of the monopole and the dipole propagations. The coupling efficiencies as functions of the wavelength are depicted in Fig. S1(e). The coupling efficiency is defined as the ratio of the transmitted power at the top surface of the gold film to the propagated power of SPPs in the +*x*- and -*x*-direction on the surface. The higher the coupling efficiency, the stronger plasmonic waves are excited by the normally incident light. It is one of the factors to be considered alongside the phase difference between the two polarizations and the total power of each polarization when the operation wavelength is selected. The wavelength of 1475 nm has been selected for detailed investigation where the E_y-field shows strong coupling.

2. *Power ratio at different wavelengths by angle and phase modulations*

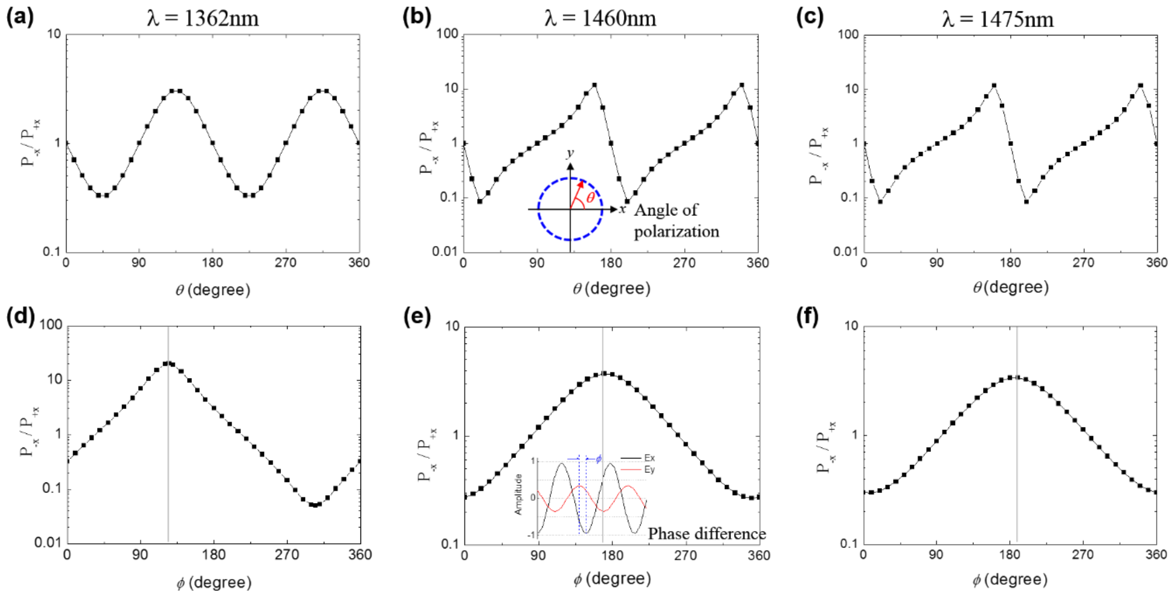

Fig. S2 Power ratios (a, b, c) as functions of the polarization angle, and (d, e, f) as functions of the phase difference. The wavelengths are (a, d) 1362 nm, (b, e) 1460 nm, and (c, f) 1475 nm.

The power ratio for three different wavelengths is examined: 1362 nm where the amplitudes of the monopole and the dipole are almost equal, 1475 nm which is the resonance wavelength of the y-polarized excitation, and 1460 nm near the resonance for comparison. The maximum power ratio which can be obtained by the incident polarization is found to depend on the wavelength. The maximum power ratio for 1362 nm is found to be ~3 at θ = 135° (Fig. S2(a)) whereas the power ratio over 10 for the wavelength of 1475 nm is obtained at θ = 160° (Fig. S2(c)). No phase difference is included in the simulations shown in Figs. S2(a), (b), and (c). As shown in Fig. S2(d-f), the power ratio can also be controlled by the phase difference between the E_x and E_y incident fields. Because the amplitudes of the monopole and the dipole are equal at 1362 nm, the maximum power ratio could be obtained just by changing the relative phase delay. However, due to the different amplitudes between the monopole and the dipole at 1475nm, the maximum power ratio obtained by controlling the phase difference is less than that by adjusting the angle of polarization. The shift of the power ratio peak from the phase difference of 120° to 180° indicates the change of the relative phase delay between the monopole and the dipole SPPs due to the off-resonance excitation.

3. *Travelling cylindrical waves*

When the equation of a cylindrical wave is given as

$$\nabla^2 U - \frac{1}{v^2}\frac{\partial^2 U}{\partial t^2} = 0$$

where *v* is the phase velocity of the wave, it is known that the amplitude $U(\rho, \phi, t)$ of the cylindrical waves is

$$U(\rho,\phi,t) = J_m(k\rho)\left(a_1 e^{im\phi} + a_2 e^{-im\phi}\right)\left(b_1 e^{i\omega t} + b_2 e^{-i\omega t}\right)$$

where J_m is the Bessel function of the first kind.[1] By applying the boundary condition of $U \sim e^{i(k\rho-\omega t)}$ for large ρ, cylindrical waves which are travelling outgoing are

$$U(\rho,\phi,t) = H_m^{(1)}(k\rho)e^{im\phi}e^{-i\omega t}$$

where $H_m^{(1)}$ is the Hankel function of the first kind. Accordingly, the amplitudes of the *E*-field of the monopole and the dipole radiations can be represented as

$$E_z^m(\rho,t) = E_0^m H_0^{(1)}(k\rho)e^{-i\omega t-\gamma\rho}$$

$$E_z^d(\rho,\phi,t) = E_0^d H_1^{(1)}(k\rho)e^{i\phi}e^{-i\omega t-\gamma\rho}$$

by assigning the azimuthal dependence *m* of 0 and 1 based on their symmetric and anti-symmetric characteristics, respectively. The amplitude of the total *E*-field is subsequently given

$$E_z(\rho,\phi,t) = E_z^m(\rho,t)e^{-i\delta} + E_z^d(\rho,\phi,t)$$

considering the temporal phase difference between the monopole and the dipole waves due to the off-resonance excitation of SPPs. The travelling waves are shown in Fig. S3. The total E_z-field with the temporal phase difference $\delta = \pi/2$ is shown in Fig. S3(c). The one-way propagation in positive *x*-direction is clearly obtained.

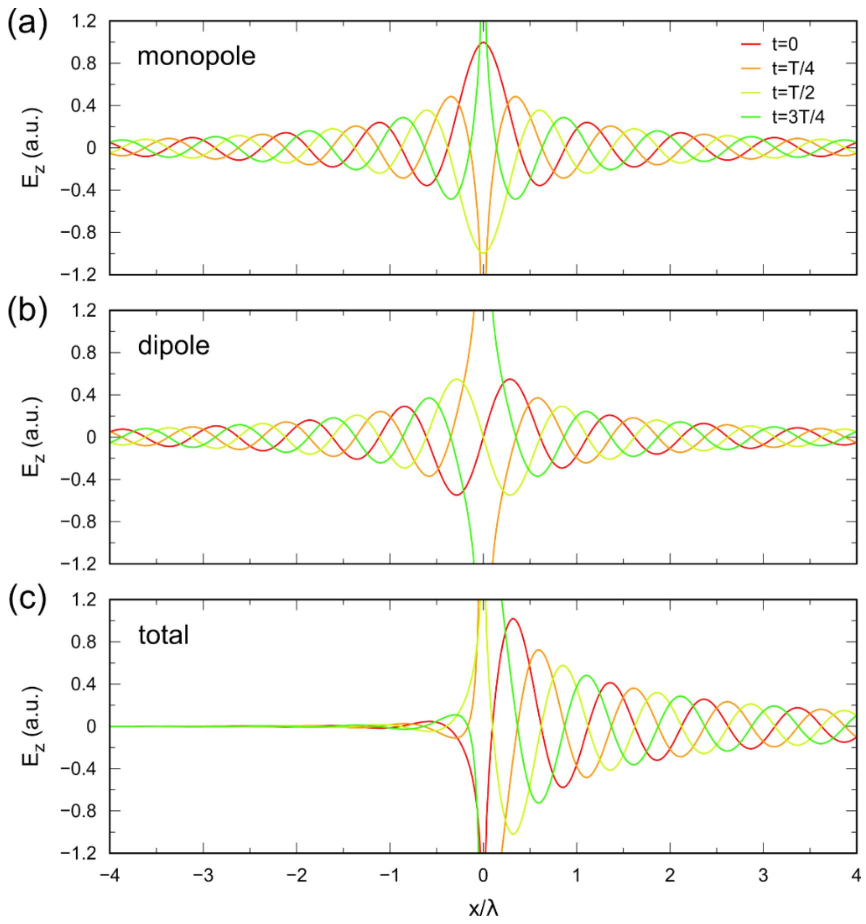

Fig. S3 The E_z-field amplitudes of the SPP waves evolving in time. T is the period. (a) Monopole and (b) dipole waves at t of 0, T/4, T/2, and 3T/4. (c) Total E_z-field when the temporal phase difference $\delta = \pi/2$. An obvious one-way propagation in positive x-direction is obtained.

4. Structural dependence

Structural dependence is studied to check the potential improvement of the proposed devices. One possibility to improve the efficiency is to bring the two resonances of the monopole and the dipole induced by E_y and E_x waves illumination. We investigated the resonance shifts by varying the radius and the gap of the CSRR. The shift to the shorter wavelength for both polarizations is observed as reducing the radius of the ring as shown in Figs. S4(a) and (c). The difference between the two resonance wavelengths decreases as the ring gets smaller. However, the decrease is not significant, and it is hard to reduce the size further due to the fabrication limit. Reducing the gap shows intriguing results as shown in Figs. S4(b) an (d). The dipole resonance experience little shift whereas the monopole red-shifts substantially by reducing the gap. This tendency suggests a strong possibility of improvement in the aspect of efficiency. Further study is required to establish a design rule and to find an optimized structure.

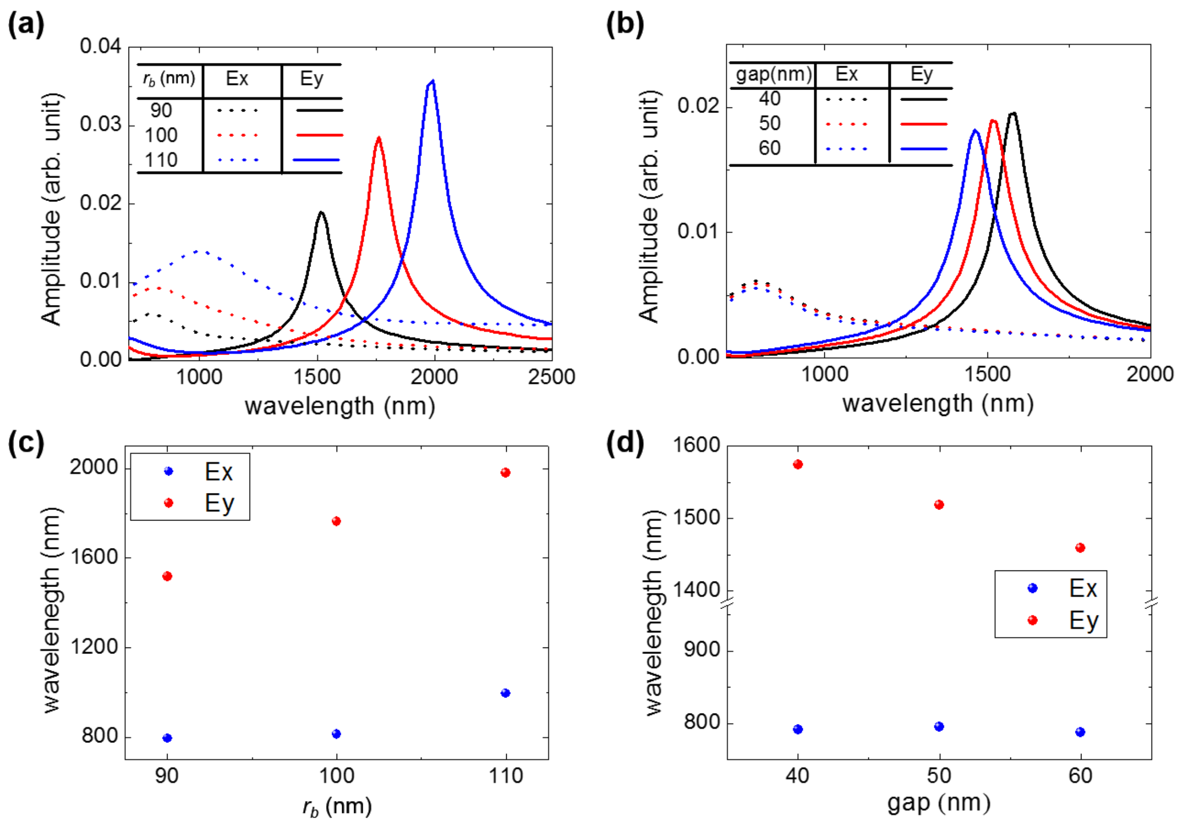

Fig. S4 Resonances depending on the structures. Spectral characteristics for different radii (a) and gaps (b). The resonant wavelengths of two incident polarizations are shown for different radii (c) and gaps (d). The gap is fixed to be 50 nm for (a) and (c), and the outer radius r_b is fixed to be 90 nm for (b) and (d). The width, $r_b - r_a$, is fixed to be 50 nm for all calculations.

References

1. Arfken, G. B. & Weber, H. J. *Mathematical Methods for Physicists, 6th Edition*. (Academic Press, 2005).
